# Examination of Rickettsial Host Range for Shuttle Vectors Based on *dnaA* and *parA* Genes from the pRM Plasmid of *Rickettsia monacensis*

**DOI:** 10.1128/aem.00210-22

**Published:** 2022-03-24

**Authors:** Nicole Y. Burkhardt, Lisa D. Price, Xin-Ru Wang, Chan C. Heu, Gerald D. Baldridge, Ulrike G. Munderloh, Timothy J. Kurtti

**Affiliations:** a University of Minnesota, Department of Entomology, Saint Paul, Minnesota, USA; University of Queensland

**Keywords:** *Rickettsia* plasmids, *Rickettsia monacensis*, *Rickettsia amblyommatis*, shuttle plasmids, transformation, host range, *Rickettsia*, plasmids, shuttle vector

## Abstract

The genus *Rickettsia* encompasses a diverse group of obligate intracellular bacteria that are highly virulent disease agents of mankind as well as symbionts of arthropods. Native plasmids of Rickettsia amblyommatis (AaR/SC) have been used as models to construct shuttle vectors for genetic manipulation of several *Rickettsia* species. Here, we report on the isolation of the complete plasmid (pRM658B) from Rickettsia monacensis IrR/Munich mutant Rmona658B and the construction of shuttle vectors based on pRM. To identify regions essential for replication, we made vectors containing the *dnaA* and *parA* genes of pRM with various portions of the region surrounding these genes and a selection reporter cassette conferring resistance to spectinomycin and expression of green fluorescent protein. Rickettsia amblyommatis (AaR/SC), *R. monacensis* (IrR/Munich), Rickettsia bellii (RML 369-C), Rickettsia parkeri (Tate’s Hell), and Rickettsia montanensis (M5/6) were successfully transformed with shuttle vectors containing pRM *parA* and *dnaA*. PCR assays targeting pRM regions not included in the vectors revealed that native pRM was retained in *R. monacensis* transformants. Determination of native pRM copy number using a plasmid-carried gene (RM_p5) in comparison to chromosomally carried *gltA* indicated reduced copy numbers in *R. monacensis* transformants. In transformed *R. monacensis* strains, native pRM and shuttle vectors with homologous *parA* and *dnaA* formed native plasmid-shuttle vector complexes. These studies provide insight on the maintenance of plasmids and shuttle vectors in rickettsiae.

**IMPORTANCE** *Rickettsia* spp. are found in a diverse array of organisms, from ticks, mites, and fleas to leeches and insects. Many are not pathogenic, but others, such as Rickettsia rickettsii and Rickettsia prowazeckii, can cause severe illness or death. Plasmids are found in a large percentage of nonpathogenic rickettsiae, but not in species that cause severe disease. Studying these plasmids can reveal their role in the biology of these bacteria, as well as the molecular mechanism whereby they are maintained and replicate in rickettsiae. Here, we describe a new series of shuttle plasmids for the transformation of rickettsiae based on *parA* and *dnaA* sequences of plasmid pRM from Rickettsia monacensis. These shuttle vectors support transformation of diverse rickettsiae, including the native host of pRM, and are useful for investigating genetic determinants that govern rickettsial virulence or their ability to function as symbionts.

## INTRODUCTION

The genus *Rickettsia* (order *Rickettsiales*, family *Rickettsiaceae*) contains obligate intracellular alphaproteobacteria with wide-ranging effects on infected invertebrates and mammals: some, such as Rickettsia tamurae subsp. *buchneri*, are avirulent arthropod symbionts, while others (for example, Rickettsia rickettsii and Rickettsia prowazekii) are highly virulent vector-borne human pathogens causing spotted fevers and typhus. Some *Rickettsia* spp. are unique among the *Rickettsiales* in harboring plasmids (1). More than 30 plasmids have been identified in a wide range of *Rickettsia* species and strains (2–6). The diversity of *parA* on rickettsial plasmids suggests that foreign plasmids have invaded rickettsiae (7) via a conjugation system (8, 9). As many as 4 distinct plasmids, each with a distinct partitioning system, can coexist in a given rickettsial strain (2, 10). Plasmids are more commonly associated with avirulent and less virulent rickettsiae and are absent in highly virulent strains (see Table S1 in the supplemental material). Rickettsial plasmids have undergone reductive evolution, and to date, virulence has not been linked to the presence of plasmids (5, 11). These features indicate that shuttle vectors modeled after rickettsial plasmids can be developed as tools to introduce foreign genes into rickettsiae, as we have demonstrated previously (12).

The discovery of low-copy-number plasmids in many *Rickettsia* spp. (1, 2, 13) spurred development of the first shuttle vectors, a fundamental advance in rickettsial transformation technologies (12, 14). Their potential has been realized by generation of transformant rickettsiae expressing selectable markers and fluorescent proteins that have enabled study of rickettsial interactions with host cells in unprecedented detail. Examples with arthropod endosymbionts include *R. tamurae* subsp. *buchneri* and Rickettsia peacockii in tick host cells (15), as well as Rickettsia bellii expressing a plasmid-encoded heterologous *rickA* gene (16). Although the typhus group rickettsiae are among those *Rickettsia* spp. that do not harbor a native plasmid, shuttle vectors have also been used to transform and study typhus group rickettsiae, including the epidemic typhus agent, *Rickettsia prowazekii*, in L929 murine fibroblast cells (17) and the endemic typhus agent, Rickettsia typhi, in CB17 SCID mice and in association with CD8 cells (18). The first-generation rickettsial shuttle vectors are now an important component of the still limited toolbox for genetic manipulation of rickettsiae (19, 20), underscoring the need for further expansion and optimization of the vectors and for the study of plasmid biology in the genus *Rickettsia*.

The design of plasmid-based bacterial transformation vectors was influenced by discovery of incompatibility, which resulted in classification of plasmids into incompatibility groups determined by their ability to be simultaneously maintained within a single host cell. Incompatibility arises when both foreign and native plasmids carry the same or a similar *parA* gene, causing both plasmids to become unstable (21, 22). *R. tamurae* subsp. *buchneri* and Rickettsia amblyommatis have multiple independent plasmids, each with a distinct *parA* gene (2, 3, 10). The role of *parA* in plasmid partitioning and incompatibility in rickettsiae remains largely unexplored and likely has practical consequences for use of rickettsial shuttle vectors. In contrast, *R. monacensis* contains only one native plasmid, pRM, and phylogenetic analysis shows it contains a *parA* gene with no significant similarity to those from 19 other rickettsial plasmids (3), although the plasmid pRAS01 from *Rickettsia asembonensis* strain NMRCii has a *parA* gene with 66% nucleotide identity to *parA* from pRM (23). The predicted amino acid sequence of ParA from pRM is most similar to ParA-like predicted proteins from Mycobacterium and *Bartonella* spp. (25 to 53% BLASTP identities). Thus, shuttle vectors from pRM should theoretically be compatible for transformation of an expanded range of rickettsiae.

Our aim is to both expand the repertoire of shuttle vectors for use in the study of *Rickettsia* spp. and improve our understanding of the mechanisms of plasmid maintenance in diverse rickettsial species. Here, we report isolation of pRM from Rickettsia monacensis in Escherichia coli and its development as a family of shuttle vectors, which were used to transform several rickettsiae, including *R*. *monacensis* and *R. amblyommatis* AaR/SC, whose pRAM plasmids have also been developed as rickettsial shuttle vectors (12). Quantitative PCR (qPCR) results indicated that introduction of the pRM shuttle vector reduced copy numbers but did not eliminate endogenous pRM in transformed *R. monacensis*. The first-generation pRAM-based shuttle vector family and second-generation pRM-based shuttle vector family carry unrelated *parA* genes and thus diversify the range of vector choices available for transformation of rickettsiae.

## RESULTS

### Identification of minimal coding sequences required for pRM shuttle vector replication and partitioning.

**FIG 1:**
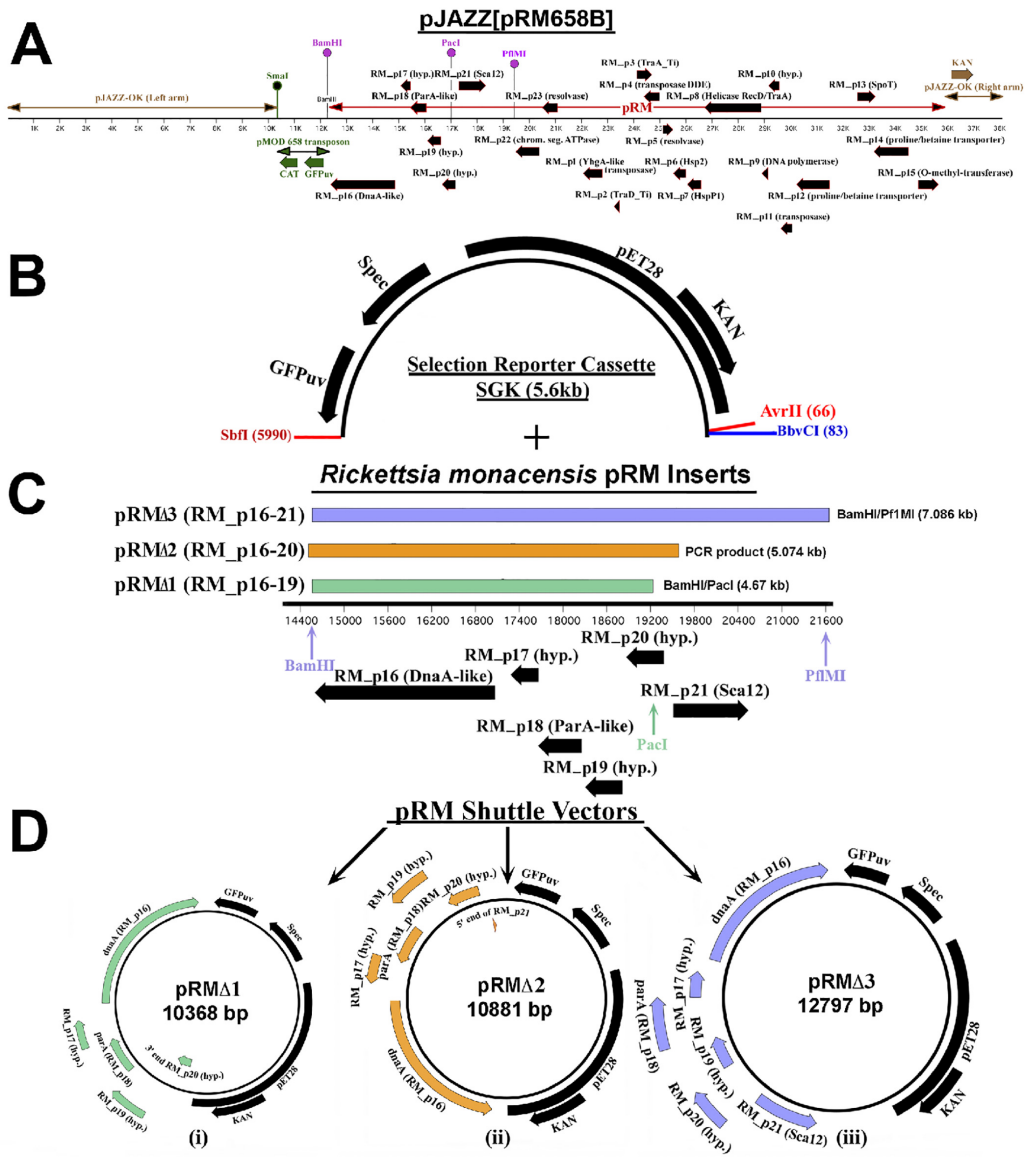
Construction of *R. monacensis* pRM shuttle vectors. (A) Schematic diagram depicting construct pJAZZ[pRM658B], the *R. monacensis* plasmid pRM with pMOD658 transposon cloned into the linear plasmid pJAZZ. The two arms formed by digested pJAZZ are indicated by brown double-ended arrows, and pRM is represented by a red double-ended arrow; genes present on pRM are denoted with black arrows. The pMOD658 transposon is shown in green; resistance to chloramphenicol (CAT) and the ability of the clone to express green fluorescent protein (GFP_uv_) are conferred by the transposon inserted in pRM. The unique SmaI site with which pRM was linearized for cloning is located in the transposon. Pink balloons indicate the restriction enzyme sites used for subcloning the *dnaA*/*parA* region of pRM. (B) The 5.6-kbp selection reporter cassette SGK was ligated with three pRM fragments of various sizes containing *dnaA* and *parA*, yielding the *R. monacensis* pRM shuttle vectors pRMΔ1, -2, and -3 (C). The numbered black line in panel C represents bp 14400 to 21600 of the 23,486-bp *R. monacensis* plasmid pRM, and the thick black horizontal arrows beneath it indicate predicted genes and their orientations. Colored vertical arrows indicate the restriction enzyme sites used during pRM subcloning, and colored bars above the numbered black line represent the three fragments of pRM contained in the finished shuttle vectors shown in panel D.

Because coding sequences that support plasmid replication and partitioning are often clustered together, we identified the minimal region required for rickettsial shuttle vector replication and partitioning by constructing three shuttle vectors with various portions of RM_p16-21 coding sequences: pRMdSGK clone 1 (pRMΔ1), pRMdSGK clone 2 (pRMΔ2), and pRMdSGK clone 3 (pRMΔ3) (Fig. 1). All three clones contained coding sequence for the pRM DnaA-like protein (RM_p16) and ParA (RM_p18), which function in DNA replication and chromosomal stability, as well as RM_p17 and RM_p19, hypothetical proteins (HPs) of unknown function, although RM_p19 contains domains with similarity to those of the HTH_XRE family transcriptional regulators. The smallest test construct, pRMΔ1, included the gene cluster RM_p16 through RM_p19 and partial coding sequence (359 bp of 507 bp) for RM_p20 (green bar in Fig. 1C), a second likely HTH_XRE family protein with 74% similarity to RM_p19. The pRMΔ2 construct extended the same *dnaA*/*parA* region through an intact RM_p20 coding sequence and an amino-terminal fragment of RM_p21 (orange bar in Fig. 1C). The pRMΔ3 construct further encoded an intact RM_p21, a likely Sca12 cell surface antigen, and approximately 1 kbp of downstream noncoding sequence (lilac bar in Fig. 1C).

### Transformation trials of five SFG *Rickettsia* spp.

The smallest test construct, pRMΔ1, transformed only *R. monacensis* (Table 1), which carries pRM as its native plasmid. In contrast, in trials with pRMΔ2 (encoding the same proteins from RM_p16 through RM_p19 as well as the intact RM_p20 XRE family protein with its upstream region) and pRMΔ3 (extended to encode the RM_p21 Sca12 cell surface antigen), both constructs transformed all five *Rickettsia* species tested (Table 1). Species successfully transformed included four spotted fever group (SFG) rickettsiae, notably *R. amblyommatis* AaR/SC, which contains 3 native plasmids, as well as *R. bellii*, which occupies a more basal phylogenetic position (24). The cluster from RM_p16 through RM_p19 thus supported transformation of the parental *R. monacensis*, but inclusion of the RM_p20 locus and/or a short upstream sequence extended the range of a pRM-based shuttle vector to other SFG and ancestral group rickettsiae. In contrast to RM_p20, RM_p21 and the associated noncoding 1-kbp sequence conferred no apparent advantage.

**TABLE 1:** Transformation of *Rickettsia* with plasmid constructs containing pRM *dnaA* and *parA*

Strain type^*a*^	Result for construct^*b*^:			
	pRAM18dRGA, 10.3 kbp	pRMΔ1 (RM_p16-*20*), 10.4 kbp	pRMΔ2 (RM_p16-*21*), 10.9 kbp	pRMΔ3 (RM_p16-21), 12.8 kbp
Strains with endogenous plasmids				
*R. monacensis* IrR/Munich (pRM, 23 kb)	+	+	+	+
*R. amblyommatis* AaR/SC (pRAM; 18, 23, and 32 kb)	+	−	+	+

Plasmid-free strains				
*R. parkeri* Oktibbeha	+	−	+	+
R. montanensis M5/6	+	−	+	+
* R. bellii* 369C	+	−	+	+

a The native plasmid name is given in parentheses, along with the approximate native plasmid size(s).

b The construct designation and size are shown, with the pRM genes included in parentheses. An italicized gene number indicates the sequence does not represent the complete gene. +, transformed; −, not transformed.

These results indicated that the loci from RM_p16 through RM_p20 of pRM contained the minimum necessary DNA sequence and protein coding capacities for a shuttle vector capable of transforming a wide range of SFG and ancestral group rickettsiae. The promoter prediction program BPROM (25) indicated a single promoter upstream of the RM_p20 locus, but none for RM_p17, -18, or -19, consistent with functional dependence of RM_p18 expression on sequences upstream of RM_p20 and a likely operon extending through the RM_p16 locus (Fig. 1D). The predicted transcription start site was at bp 19395 of pRM, with a −10 box from 19402 to 19410 and a −35 box from 19425 to 19430. A comparison of growth rates of wild-type (WT) *R. monacensis* and pRMΔ2-transformed *R. monacensis* using three separate growth curve analyses indicated no significant differences between the growth rates of WT and transformants (doubling times of 17.5 and 18 h, respectively).

### Evaluation of GFP expression in wild-type and transformed *R. monacensis* using confocal microscopy.

**FIG 2:**
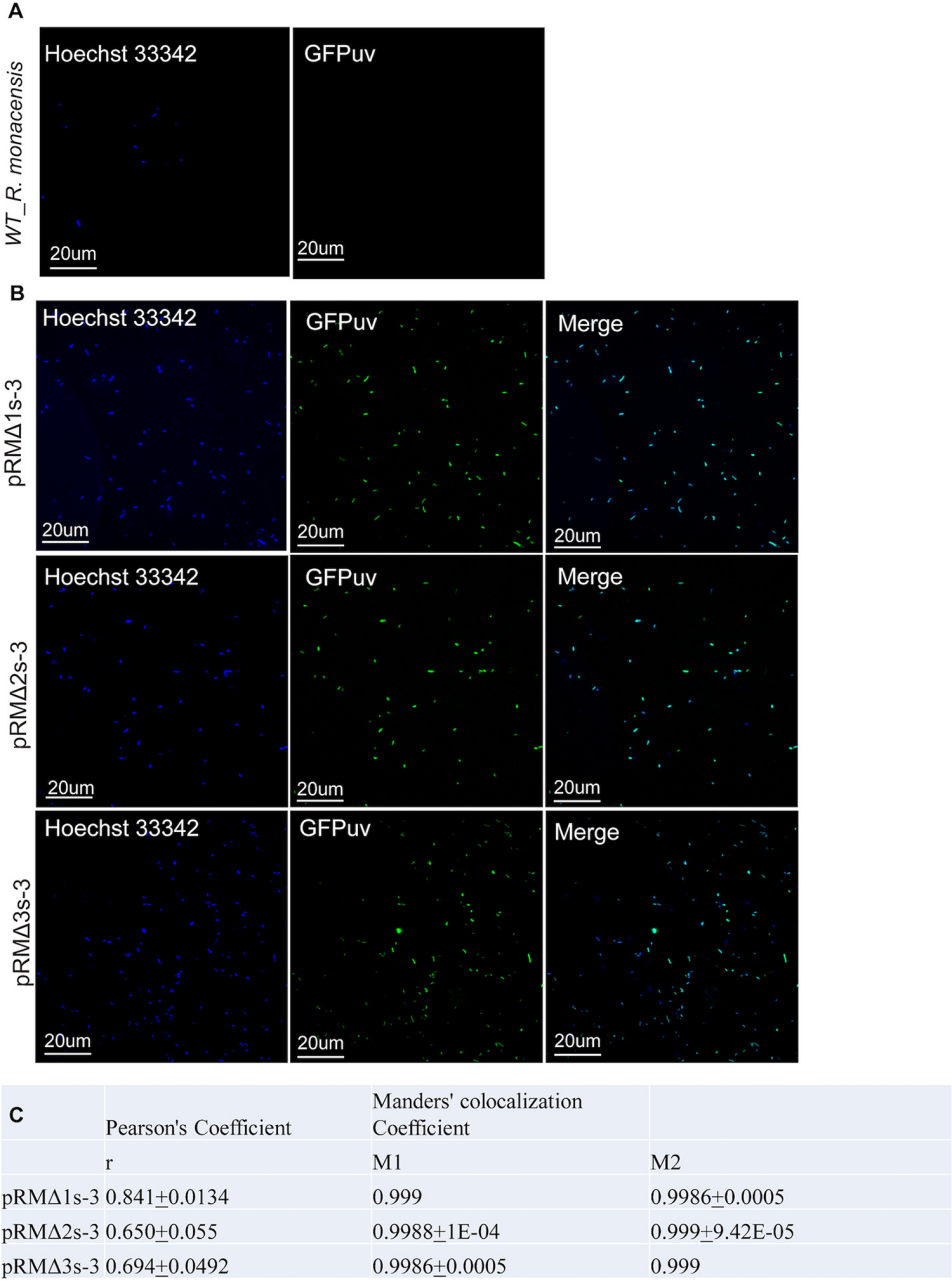
Evaluation of GFP_uv_ expression in WT and transformed *R. monacensis* using confocal microscopy. (A) WT and (B) pRMΔ1-, -2-, and -3-transformed *R. monacensis*. The first column shows fluorescent staining of rickettsial DNA (blue signal, Hoechst 33342), and the second column shows *gfp_uv_* fluorescence (green signal). The third column shows combined Hoechst 33342 and *gfp_uv_* signals of the merged fields. (C) Table showing Pearson’s correlation coefficient (PCC) and Manders’ colocalization coefficient (MCC) of the same fields of view shown in panel B to quantify colocalization of *gfp_uv_* fluorescence from transformed *R. monacensis* and fluorescence from stained rickettsial DNA. M1, fraction of blue fluorescence in areas with green fluorescence; M2, fraction of green fluorescence in areas with blue fluorescence.

The cell-free *R. monacensis* WT strain and transformants were stained with NucBlue Live Cell Stain ReadyProbes (Thermo Fisher Scientific, Waltham, MA) to visualize all rickettsiae, whether transformed or not, then gently mounted onto slides by using a Cytospin centrifuge (Thermo Fisher) and observed by confocal microscopy. The left column of Fig. 2A with a DAPI (4′,6-diamidino-2-phenylindole) filter shows all the rickettsiae present, while the middle column shows only *gfp_uv_*-expressing transformed rickettsiae (fluorescein isothiocyanate [FITC] filter). The third column (Fig. 2B) illustrates the overlap of fluorescence in the DAPI and FITC fields. Visually, the degree of colocalization indicated that nearly all the rickettsiae present were transformed. To confirm these observations, we used Pearson’s coefficient (PCC) and Manders’ colocalization coefficient (MCC) (26, 27) to evaluate the colocalization of the DAPI and FITC fluorescence. For the PCC analysis, the correlation coefficient was measured on all pixels in an individual image for three random fields. The PCC assay values range from −1 to 1, with 1 representing 100% colocalization; the PCC values are lower than would be indicated by visual analysis as low fluorescence values limit the program’s ability to evaluate and interpret pixels. The MCC assay measures the proportion of rickettsial DNA fluorescence that localizes with transformed plasmid fluorescence (M1) and vice versa (M2). Almost 100% colocalization is indicated by the very similar M1 and M2 values calculated for each of the three transformed *R. monacensis* strains (Fig. 2C).

### Presence of shuttle vector in pRM-transformed *R. amblyommatis* and *R. parkeri* and conservation of native plasmids in transformed *R. amblyommatis*.

**FIG 3:**
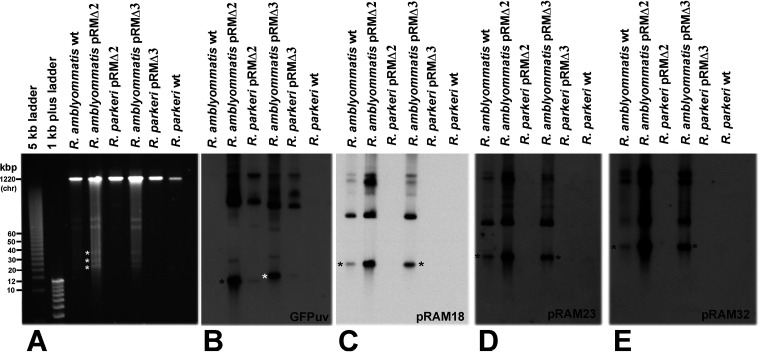
Pulsed-field gel electrophoresis and Southern blot analysis of *R. amblyommatis* and *R. parkeri* transformed with pRM shuttle vectors pRMΔ2 and pRMΔ3. *gfp_uv_* and pRAM digoxigenin-labeled probes were used to detect the presence of shuttle vector and/or plasmid pRAM18, pRAM23, or pRAM32 in transformants. (A) PFGE gel. White asterisks mark the nicked linear forms of pRAM18, pRAM23, and pRAM32, respectively. (B) Southern blot analysis of pulsed-field gel hybridized with digoxigenin-labeled *gfp_uv_* probe. The nicked linear forms of pRMΔ2 and pRMΔ3 are denoted by black and white asterisks, respectively. Higher-molecular-weight bands are supercoiled and multimer forms of the shuttle vectors pRMΔ2 and pRMΔ3. Note the absence of labeling in the WT *R. amblyommatis* and *R. parkeri* lanes. (C) Southern blot analysis of panel A hybridized with digoxigenin-labeled pRAM18 probe (invertase). (D) Southern blot analysis of panel A hybridized with digoxigenin-labeled pRAM23 probe (Hsp2). (E) Southern blot analysis of panel A hybridized with digoxigenin-labeled pRAM32 probe (RecD). Black asterisks in panels C, D, and E denote nicked linear forms of pRAM18, -23, and -32, respectively.

Undigested DNA from *R. amblyommatis* and Rickettsia parkeri transformed with pRMΔ2 and pRMΔ3 was separated by pulsed-field gel electrophoresis (PFGE) (Fig. 3A) and transferred onto Zeta-Probe membranes. Because *R. parkeri* does not contain native plasmids, hybridization of *R. parkeri* pRMΔ2 and pRMΔ3 transformants with digoxigenin-labeled *gfp_uv_* probe (28) identified the unaltered shuttle vectors, with asterisks indicating the nicked linear forms of pRMΔ2 and pRMΔ3 at 10.81 and 12.814 kbp, respectively (Fig. 3B). The *R. amblyommatis* transformants show a band pattern similar to that of *R. parkeri*; however, extra bands were present in both *R. amblyommatis* pRMΔ2 and pRMΔ3 hybridized with the *gfp_uv_* probe (Fig. 3B). Stripped Southern blots were hybridized with recombinase, *hsp2*, and helicase digoxigenin-labeled probes specific for *R. amblyommatis* strain AaR/SC plasmids pRAM18, pRAM23, and pRAM32, respectively (12). Bands of the appropriate size were observed for all 3 native pRAM plasmids (pRAM18, 18.344 kbp; pRAM23, 22.852 kbp; and pRAM32, 31.972 kbp) in the transformed *R. amblyommatis* (marked with asterisks in Fig. 3A, C, D, and E), but not in *R. parkeri*. Thus, pRM shuttle vector and all three native *R. amblyommatis* plasmids are conserved in the pRM-transformed *R. amblyommatis*.

### Testing for the presence of native pRM and shuttle vectors in pRMΔ-transformed *R. monacensis*.

**FIG 4:**
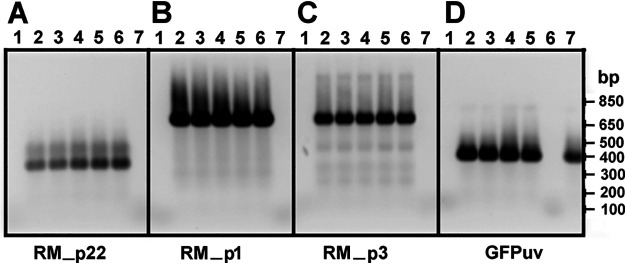
PCR results demonstrating the presence of native pRM in pRMΔ-transformed *R. monacensis*. PCR amplicons were electrophoresed on a 1% agarose gel stained with GelGreen (Biotium, Hayward CA). (A, B, and C) PCR results using primers specific for native pRM genes (RM_p22, RM_p1, and RM_p3) not present in pRM shuttle vectors. (D) PCR products using *gfp_uv_*-specific primers (dGFPuvF2/dGFPuvR2) to detect shuttle vector. Lane designations: 1, no-template control; 2, pRMΔ2 passage 1 transformant; 3, pRMΔ2 passage 3 transformant; 4, pRMΔ1 passage 3 transformant; 5, pRMΔ3 passage 3 transformant; 6, WT *R. monacensis*; 7, *R. amblyommatis* pRMΔ2 transformant.

To assess native pRM in shuttle vector transformants, genomic DNA from WT and transformant *R. monacensis* strains was PCR amplified with native pRM-specific primer sets (Table 2). All three *R. monacensis* transformants contained native pRM, as indicated by the presence of amplicons from genes present in native pRM but not in the shuttle vectors (Fig. 4A, B, and C). The dGFPuvF2/R2 primer amplicons confirmed the presence of the shuttle vectors (Fig. 4D). *R. amblyommatis* AaR/SC pRMΔ2 DNA was used as a control to confirm primer specificity; as expected, it yielded no amplicon with native pRM primer sets but was positive with dGFPuvF2/R2 primers.

**TABLE 2:** PCR and qPCR primers used to distinguish native pRM and shuttle vectors and estimate their copy numbers

Primer	Primer ID	Sequence	Amplicon size (bp)
RM_p22 specific	RM_p22 F	TCTACACGAGGCGTTATTCTTTCC	335
	RM_p22 R	CTCAATCTGGTCTTGCGAGAGC	
RM_p1 specific	RM_p[pre1]F	CTAAAGTAGCAGGTGTGCCATCG	676
	RM_p1R	ATCACTGGGAACAACAAGGGG	
RM_p3 specific	RM_p[pre3]F	TCTGATTTTGGTTTGCTGGGC	677
	RM_p3R	CATTCTGTCCTTGGCTCCTTTG	
Shuttle vector specific	dGFPuvF2	TTCTGTCAGTGGAGAGGGTGAAGGT	399
	dGFPuvR2	CCATTCTTTTGTTTGTCTGCCGTG	
Citrate synthase qPCR^*a*^	qCS-F	TCGCAAATGTTCACGGTACTTT	74
	qCS-R	TCGTGCATTTCTTTCCATTGTG	
GFPuv qPCR^*b*^	qGFPuvF	CAGTGGAGAGGGTGAAGGTGATGC	114
	qGFPuvR	ACCATAAGAGAAAGTAGTGACAAGTGTTGGC	
RM_p5 qPCR	qRM_p5F	CAGCATCAGGAGAGTTGGTATT	106
	qRM_p5R	CGAATACGTGCAGCACTAAGA	

a See reference 28 for details.

b See reference 12 for details.

### Copy number ratios of native pRM and pRM shuttle vectors in transformed *R. monacensis*.

**FIG 5:**
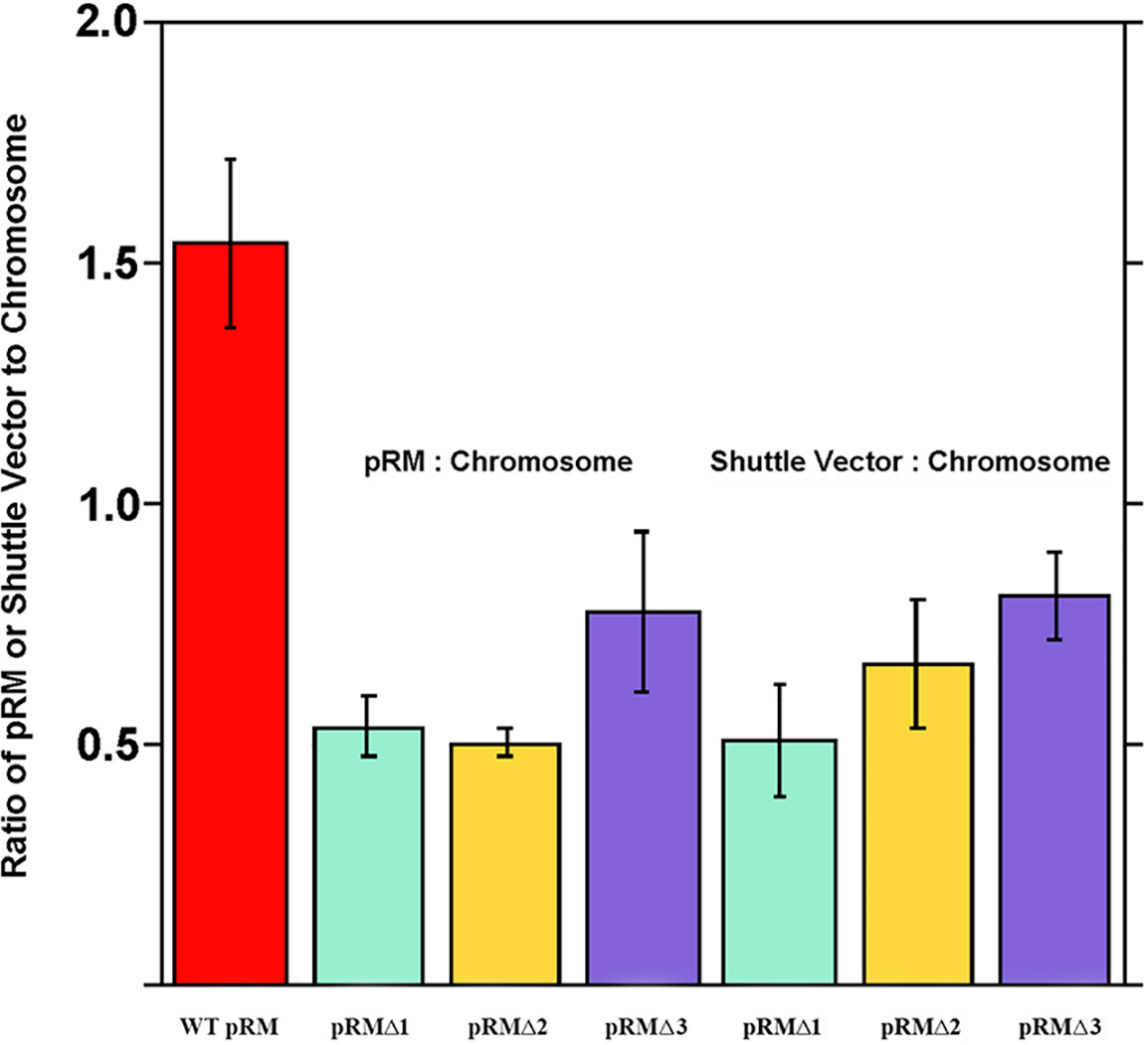
Plasmid-to-chromosome copy number ratios in transformed *R. monacensis.* Native plasmid (pRM), shuttle vector, and chromosomal copy numbers were quantified by qPCR of RM_p5, *gfp_uv_* and *gltA,* respectively. Passage numbers (e.g., p17) used for this assay were as follows: WT, p17 (all 3 replications); pRMΔ2, p10 (all 3 replications); pRMΔ1, one replication with p3 and two replications with p9; pRMΔ3, one replication with p3 and two replications with p7.

Quantitative PCR (qPCR) estimation of the relative ratio of the single-copy pRM-encoded resolvase RM_p5 (qRM_p5F/qRM_p5R primer set in Table 2) and chromosome-carried *gltA* genes (29) indicated 1.5 copies of pRM for each chromosome copy in WT *R. monacensis*, which decreased to a ratio of 0.5 to 0.8 in pRMΔ1-, -2-, and -3-transformed *R. monacensis* (Fig. 5). This ratio was mirrored by the 0.5-to-0.9 ratio of shuttle vector (qGFPuvF/qGFPuvR primers from shuttle vector-carried *gfp_uv_* in Table 2) to chromosome in the transformant rickettsiae (Fig. 5). There were no further significant changes in copy number ratios during serial passage of the transformant rickettsia (up to 20 passages in the case of pRMΔ2) (data not shown).

### Presence of shuttle vector and native plasmid complexes in pRM-transformed *R. monacensis*.

**FIG 6:**
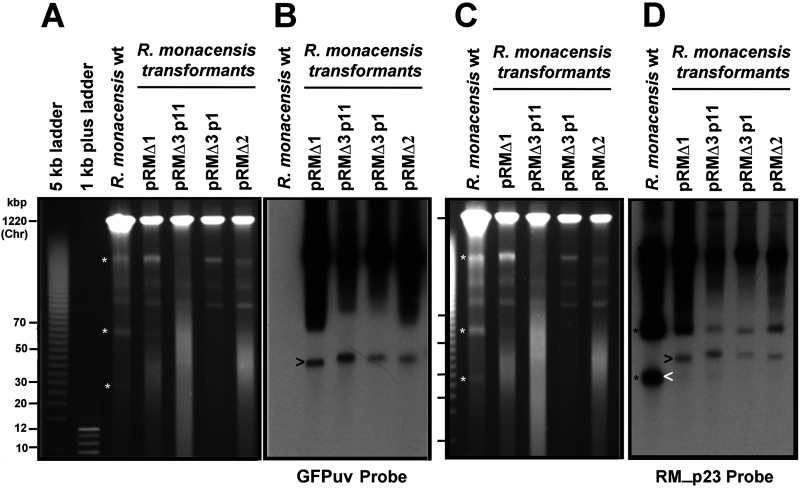
Pulsed-field gel electrophoresis and Southern blot analysis of *R. monacensis* pRM shuttle vector transformants. (A) PFGE gel and (B) Southern blot analysis of panel A hybridized with digoxigenin-labeled *gfp_uv_* probe showing the presence of shuttle vectors (the black arrowhead indicates nicked linear forms). (C) PFGE gel and (D) Southern blot analysis of panel C hybridized with digoxigenin-labeled RM_p23 probe showing the presence of native pRM in the WT *R. monacensis* (the white arrowhead indicates the nicked linear form) and a larger form (black arrowhead) in each of the transformants that matches the size of the products that hybridized with the *gfp_uv_* probe (B). White asterisks in panels A and C mark the 3 main forms of native pRM on the PFGE gels, while the black asterisks show corresponding hybridization of said forms with RM_p23 probe in panel D. p1 and p11 refer to the passage numbers of pRMΔ3.

To confirm the PCR-indicated presence of native pRM in pRMΔ shuttle vector-transformed *R. monacensis*, undigested genomic DNA from WT and pRMΔ1, -2, and -3 transformants was separated by pulsed-field gel electrophoresis (Fig. 6) and transferred onto Zeta-Probe membranes. Nicked linear, circular, and multimeric forms of pRM were present in the WT *R. monacensis* lanes (Fig. 6A and C, bands with white asterisks). The presence of the shuttle vector in *R. monacensis* transformed with pRMΔ1, pRMΔ2, and pRMΔ3 was demonstrated by hybridization with digoxigenin-labeled *gfp_uv_* probe (absent in the WT) (Fig. 6B). The *gfp_uv_* probe localized to bands in the ∼40-kbp region (arrowhead) rather than at 10 to 12 kbp (the size of the shuttle vectors), indicating that there are no detectable monomeric pRMΔ1, pRMΔ2, or pRMΔ3 in transformant populations.

The PFGE gel shown in Fig. 6C was transferred to a membrane and hybridized with a probe containing digoxigenin-labeled RM_p23 (13), a gene present on native pRM but absent on the shuttle vectors (Fig. 6D). In the WT lane, the probe hybridized to the 20- to 30-kbp linear nicked and multimeric forms (black asterisks) of pRM. In the transformant lanes, the RM_p23 probe hybridized in the ∼40-kbp region (Fig. 6D, arrowhead) at the same relative positions as the *gfp_uv_* probe (Fig. 6B). The strong hybridization of the RM_p23 probe at the 40-kbp position in transformant rickettsia lanes versus weak hybridization at the lowest position (lowest black asterisk in panel D) indicates the likely presence of native pRM that is predominantly complexed with shuttle vector, as transformants lacking pRM would not hybridize with RM_p23.

## DISCUSSION

Although plasmids have been extensively studied in other bacteria, the discovery of plasmids in rickettsiae is fairly recent (1, 2, 13), and their biological function is largely unexplored. Genomic analysis has shown that rickettsiae have undergone reductive evolution (30–33), resulting in such characteristics as AT enrichment, high conservation of genome sequences among species, higher levels of virulence, and variable presence and numbers of plasmids (3). Rickettsial plasmids have likewise undergone reductive evolution and mirror rickettsial genomes in relative size and GC content (34). It has been proposed that a rickettsial ancestor supported a plasmid system that was lost in some species due to their unique obligate intracellular life cycle (3, 34), but other species presumably retained plasmids due to an advantage conferred by their presence. Plasmids are known agents of horizontal gene transfer, facilitating host adaptation/virulence, antibiotic and stress resistance, and genetic plasticity (1, 34–36). Although the origins and functions of many rickettsial plasmid gene sequences have been inferred from similarities to those of other bacteria, the role of plasmids and their interactions in rickettsiae remain to be elucidated. Real-time PCR and whole-genome sequencing showed that these are low-copy-number plasmids (2), while creation of shuttle vectors from pRAM18 and pRAM32 and their subsequent transformation into rickettsiae confirmed that *parA* and *dnaA* were essential for plasmid replication and maintenance (12). However, the mechanism by which these genes function is yet to be identified.

We have developed two new *Rickettsia* plasmid shuttle vectors, pRMΔ2 and pRMΔ3, that can be used to transform plasmid-free *Rickettsia* spp. as well as those carrying native plasmids. In conjunction with pRMΔ1, they collectively contained various regions of pRM surrounding the *dnaA* and *parA* genes. Analysis of their relative efficacies in transformed rickettsiae allowed us to identify plasmid sequences important for the replication and maintenance of the shuttle vectors in rickettsiae. Our data indicated that RM_p20 or its immediate upstream sequence was required for shuttle vector replication in rickettsiae. Specifically, *R. parkeri*, *R. bellii*, Rickettsia montanensis (plasmid free), and *R. amblyommatis* strain AaR/SC (carrying 3 plasmids) were not transformed with shuttle vector pRMΔ1 (Fig. 1C, containing RM_p16 through RM_p19 and the 3′ end of RM_p20) but were transformed with shuttle vectors pRMΔ2 and Δ3 (containing RM_p16 through RM_p20 and pRM21, respectively). These data support the prediction (37) that the region of pRM containing RM_p17 to RM_p20 forms an operon. It is possible that RM_p19 and -20 are not themselves required because absence of a single promoter upstream of RM_p20, as predicted by BPROM (25), would prevent expression of RM_p18 (*parA*) in pRMΔ1. Without expression of the ParA chromosomal stability protein, the shuttle vector would not be maintained, consistent with the observed phenotype. In contrast, *R. monacensis* was transformed with pRMΔ1 when the other species were not, likely due to the presence of native pRM providing the necessary ParA for maintenance of the shuttle vector.

Partition systems usually include three features: a site that acts like a centromere, a centromere-binding protein (CBP) (usually encoded by *parB*), and an NTPase (*parA*) (38). As none of the genes in the pRM operon have similarity to known *parB* genes, the pRM partitioning system could work in one of several ways. The partitioning system could rely on the chromosomal *parB* to work or represent a new type of system, as in R388, which functions with a single protein and a centromere site (38). Alternatively, the pRM hypothetical genes could act in the capacity of *parB* as CPBs are not necessarily significantly similar in sequence but are typically dimers of helix-turn-helix (HTH) or ribbon helix-helix DNA-binding proteins (38). Both RM_p19 and -20 have HTH_XRE domains and might function as CPBs.

The pRM operon containing *parA* appears to be unique among known rickettsial plasmids. A BLASTN search of the genes RM_p17 and -20 showed no rickettsial homology, while RM_p19 only had homology with the rickettsial endosymbiont of Ixodes pacificus plasmid (pREIP). Interestingly, translation of the nucleotide sequence for RM_p19 and -20 and subsequent BLASTP of the amino acid sequence shows a low level of similarity to pREIP (36% identity and 59% positive for RM_p19; 40% identity and 60% positive for RM_p20) and to rickettsial endosymbionts of a variety of arthropods, including beetles such as Platyusa sonomae and *Bembidion* nr Transversale, bedbugs (Cimex lectularis), and midges (Culicoides impunctatus). These Torix clade *Rickettsia* spp. (39) have 2 or 3 different regions of similarity for both RM_p19 and -20 and have identities ranging from 30 to 40%, with 53% to 62% positive.

The ParA protein encoded on *R. monacensis* pRM is sufficiently distinct from those of pRAM18, -23, and -32 (the native plasmids of *R. amblyommatis*) that it should support uptake and maintenance of the pRM shuttle vector in *R. amblyommatis*. In their comparison of rickettsial plasmid genes, El Karkouri et al. (3) showed that pRM *parA* was distinct from the *parA* of all other sequenced rickettsial plasmids. A BLASTP search of the ParA protein from pRM identified only one rickettsial homolog (R. asembonensis, with 46% identity), while its closest match was from a *Mycoplasmataceae* bacterium, with 53% identity. Lack of interactions between nonhomologous ParA proteins may explain why *R. amblyommatis* strain AaR/SC was transformed with pRM shuttle vectors, despite its three native plasmids and their potential for causing plasmid incompatibility. On the other hand, the successful transformation of *R. monacensis* with its own pRM shuttle vectors was unexpected. It is possible that the presence of identical ParAs from native plasmids and shuttle vectors supports mutual plasmid maintenance in rickettsiae rather than promoting incompatibility. These results also highlight the fact that plasmid transformation of different *Rickettsia* species is by no means a routine activity with universally predictable results. For example, the construct pRAM18/Rif/GFPuv, containing full-length pRAM18, successfully transformed *R. bellii* and *R. parkeri* (12), but for unknown reasons, shuttle vectors that incorporated full-length pRM instead of pRAM18 (data not shown) were unable to transform R. montanensis, *R. monacensis*, *R. peacockii*, and *R. parkeri* or 3 strains of *R. amblyommatis*. Thus, there is still much to learn about the role of these rickettsial plasmids in the functioning of rickettsiae and the mechanisms by which they operate.

The studies reported here give us a better understanding of the mechanisms of rickettsial plasmid maintenance in diverse rickettsial species. We explored the basis for the ability of pRM-based shuttle vectors to transform *R. monacensis*. Our PCR results detected the presence of shuttle vector in transformants, which continued to persist through serial transferring. They also indicated a decrease in the ratio of pRM to the chromosome, suggesting that transformants harbored an average of two or more chromosome copies per cell or that plasmids were cured from some of the rickettsiae. Nevertheless, data from Fig. 2 suggest that almost 100% of the rickettsiae contained the shuttle vector. Furthermore, the results showed that the use of homologous rickettsial *parA* regions leads to the formation and maintenance of complexes between shuttle vectors and native plasmids, suggesting possible defects in partitioning of plasmids carrying the same *parA* genes. The surprising ability of *R. monacensis* to be transformed by shuttle vectors containing the *parA* gene from its native plasmid emphasizes the need for further study of plasmid maintenance and incompatibility in rickettsiae, and further studies are needed to elucidate the molecular basis for the apparent linkage of shuttle vectors with pRM present in *R. monacensis*.

## MATERIALS AND METHODS

### Bacterial strains and plasmids.

The Rickettsia monacensis strain IrR/Munich^T^ (40) WT was used at passages 10 to 70 after initial isolation from a tick. It was propagated in Ixodes scapularis cells, line ISE6, as described previously (16). *R. amblyommatis* (strain AaR/SC), *R. bellii* (strain RML 369-C), *R. parkeri* (strain Tate’s Hell), and R. montanensis (strain M5/6) were propagated in the same manner as *R. monacensis*.

The *R. monacensis* plasmid pRM was cloned in E. coli as previously described (37). Briefly, electroporation of *R. monacensis* with the pMOD658 transposon yielded the Rmona658B transformant, in which the pMOD658 transposon encoding chloramphenicol acetyltransferase (CAT) and carrying a *gfp_uv_* fluorescent marker was inserted into pRM (37). The mutated pRM was cloned in its entirety by chloramphenicol marker rescue of Big Easy TSA E. coli cells (Lucigen, Middleton, WI) electroporated with Rmona658B genomic DNA that had been linearized by digestion with SmaI and ligated into the linear vector pJAZZ (Lucigen). The resulting construct was termed pJAZZ[pRM658B] (Fig. 1A).

The following abbreviations are used to designate plasmids and their derivatives: (i) “S” indicates spectinomycin/streptomycin resistance conferred by the aminoglycoside adenyltransferase gene, *aadA*, driven by a rickettsial *ompA* promoter, (ii) “G” indicates green fluorescent protein (GFP) encoded by *gfp_uv_* under regulation of the rickettsial *ompA* gene promoter, and (iii) “K” indicates kanamycin resistance from the pET-28a(+) vector (Novagen, EMD Millipore, Bedford, MA), adapted as described below. Specific nucleotide spans from pRM are from accession no. EF564599 (37). Multiple cloning sites (MCSs) are designated “[MCS].” Promoters are designated “*p*.”

### Construction of pRM-based shuttle vectors. (i) Preparation of selection reporter cassette.

We constructed a cassette into which pRM fragments could be cloned. The SGK selection reporter cassette (see Fig. S1C in the supplemental material) was derived from the previously constructed shuttle vector pRAM18dRGA[MCS] (12) (Fig. S1A) and contained genes needed for replication and antibiotic selection in E. coli as well as reporter and antibiotic selection genes for use in rickettsiae (Fig. S1C). Replacement of pGEM with a 3.159-kbp DraIII/PshA fragment of the pET-28a(+) vector was prompted by previous experiments indicating that some genes cloned into the pRAM18 shuttle vector MCS were less stable in pGEM than pET. Because spectinomycin and streptomycin are water soluble and not used to treat rickettsioses, but rifampin may be, we replaced the *rpsLp-arr-2_Rp_* (RIF)/*ompAp*-*gfp_uv_* cassette with an *ompAp-aadA*/*ompAp*-*gfp_uv_* cassette (see Fig. S2B in the supplemental material).

### (ii) Subcloning pRM.

To identify which region(s) of pRM yielded the most effective shuttle vector for rickettsial transformation, we cloned specific fragments of pRM to create a family of deletion constructs: pRMΔ1, pRMΔ2, and pRMΔ3. To construct pRMΔ1 and pRMΔ3, pJAZZ[pRM658B] was digested with BamHI/PacI or BamHI/PflMI (Fig. 1A and B), and the desired 4,671-bp and 7,177-bp fragments containing either RM_p16 to -20 (pRM bp 14561 to 19231) or RM_p16 through -21 (pRM bp 14561 to 21646) were gel purified (Zymoclean Gel DNA Recovery kit; Zymo Research, Irvine, CA). The fragment ends were blunted (DNATerminator) and ligated into the blunt and dephosphorylated selection reporter cassette (SGK), creating the 10,368-bp pRMΔ1 and 12,797-bp pRMΔ3, respectively (Fig. 1D). To construct pRMΔ2, Q5 DNA polymerase (New England Biolabs) was used to PCR amplify RM_p16 through RM-p20 and into the 5′ end of RM_p21 (pRM bp 14512 through 19585) with primers RM_p16 FOR NheI/RM_p21Rev NheI (5′-TAT TGC TAG CCG TAA GGA ACA GTT GGT GAG-3′ and 5′-ATA TGC TAG CGT TAA TAT GCC TCG GGC TAC-3′). The PCR product with NheI sites at both ends (Fig. 1C) was incubated with *Taq* polymerase to create A′ overhangs and cloned into pCR4 with the TOPO TA Cloning kit (Invitrogen, Carlsbad, CA). Clones were sequenced to verify that they contained the correct pRM fragment, which was then recovered by restriction digest with NheI and ligated it into dephosphorylated, NheI-digested SGK, to yield the 10,881-bp shuttle vector pRMΔ2 (Fig. 1D).

To predict the location of rickettsial promoters in the pRM fragments cloned into the shuttle vectors, we used BPROM (http://www.softberry.com/berry.phtml?topic=bprom&group=programs&subgroup=gfindb) (25).

### Preparation of shuttle vector plasmid DNA.

Endotoxin-free maxipreps (Qiagen, Valencia, CA) were prepared for all pRMΔ shuttle vectors as per the manufacturer’s recommendations, for use in transforming rickettsiae. The integrity and orientation of inserted genes were reconfirmed by sequencing fragment junctions and by restriction digest analysis. (See Table 3 for the sequencing primers.)

**TABLE 3:** Sequencing primers for shuttle vector pRMΔ2

Primer ID	Sequence	pRM coordinates
RM_p[16-20] seq F1	5′-TCTTTATTTGTCGCTCGC-3′	bp 15048–15065
RM_p[16-20] seq F2	5′-TCCTGGATTGTCTTCAAG-3′	bp 15655–15672
RM_p[16-20] seq F3	5′-TTAGCCTTATCCATACCG-3′	bp 16484–16501
RM_p[16-20] seq F4	5′-CGTGGTGATAATGAGGAGTC-3′	bp 16787–16806
RM_p[16-20] seq F5	5′-TACGCCACTATTCCGCTG-3′	bp 17415–17432
RM_p[16-20] seq F6	5′-AAGATAATCCAGCACCAG-3′	bp 18018–18035
RM_p[16-20] seq F7	5′-GATTTTCCTACACCACCC-3′	bp 18209–18226
RM_p[16-20] seq F8	5′-CAGGAGTGGTAAGAGTCCTT-3′	bp 18958–18977

### Transformation of rickettsiae.

Rickettsiae were purified and electroporated as described previously (16). Rickettsial transformants were selected using growth medium containing spectinomycin and streptomycin, each at a final concentration of 100 μg/mL. Cultures were monitored for expression of the GFP_uv_ reporter on a weekly basis by using an inverted microscope (Nikon Diaphot TMD with Y-FL-epifluorescence attachment and a sapphire GFP 31043 filter) or by examining wet mounts on an upright Nikon Eclipse E400 microscope with a B-2E/C FITC filter (Nikon, Melville, NY).

### Growth rate analysis of *R. monacensis* transformed with pRMΔ2.

Multiwell plates of ISE6 cells were inoculated with *R. monacensis* WT at a 1:25 dilution and sampled at selected times (2.8, 48.5, 72.8, 96.4, 119.7, 143.7, 168.9, and 191.4 h postinoculation [hpi]). Two plates of the *R. monacensis* WT diluted 1:50 were sampled at 0, 22.8, 47.2, 70.3, 94.5, 119.8, 137.7, and 167 hpi. One plate of ISE6 cells inoculated with the *R. monacensis* pRMΔ2 transformant diluted 1:25 was sampled at 4, 49.5, 97.2, 145.3, 215.5, 264.2, 311.5, and 359.5 hpi. Two additional plates seeded with the same transformant were diluted 1:10 and 1:25 and sampled at 3.3, 72.1, 98.5, 121.2, 145, 167.2, 217.8, and 265.4 hpi. Growth rates were based on qPCR-determined levels of chromosomal single-copy gene *gltA* (29) from the average of 3 growth curve analyses.

### Evaluation of GFP expression in WT and transformed *R. monacensis* using confocal microscopy.

Cell-free WT and transformed *R. monacensis* cells were resuspended in complete medium and incubated with NucBlue Live Cell Stain ReadyProbes reagent (1: 50 dilution) (Thermo Fisher Scientific) in the dark for 30 min at room temperature. Fifty-microliter aliquots of cell-free *R. monacensis* were deposited onto microscope slides (Cytospin centrifuge; Thermo Fisher) at 200 rpm for 3 min. The slides were mounted with 3 μL 1× phosphate-buffered saline (PBS) and imaged on an Olympus BX61 DSU confocal microscope with a 60× objective via a double-wavelength filter (DAPI, excitation at 365 nm and emission at 480 nm; FITC, excitation at 495 nm and emission at 519 nm). Colocalization of fluorescence from *gfp_uv_* (transformed *R. monacensis*) and NucBlue (rickettsial DNA) was analyzed by determining signal overlap for each of three random fields of view using Image Fiji (with the JaCoP plugin and Co-localization Threshold plugin), Pearson’s coefficient (PCC), and calculation of Manders’ colocalization coefficients (MCCs) (26, 27).

### Pulsed-field gel electrophoresis and Southern blot analyses.

Concentrations of cell-free rickettsiae were estimated using optical density at 600-nm (OD_600_) values. Approximately 1.0 × 10^9^ to 2.0 × 10^9^ bacteria were embedded in 1% Incert agarose (BMA, Rockland, ME), and rickettsial DNA was released as previously described, except incubations were performed at 50°C (37). Agarose plugs were equilibrated with 0.5× Tris-borate-EDTA (TBE), and 1/2 of each plug was inserted into a well of a 1% LE (low-electroendosmosis) agarose gel (Beckman Instruments, Inc., Palo Alto, CA) and electrophoresed on a CHEF Mapper XA pulsed-field gel electrophoresis system (Bio-Rad, Hercules, CA) as previously described, except the setting for “molecular weight low” was 5 kbp (12). Depurinated gels were transferred onto Zeta-Probe GT genomic membranes (Bio-Rad) and hybridized (50°C), washed, labeled, detected, stripped, and rehybridized as previously described (12, 13, 28, 37). The DNA probes used were specific for genes encoding GFP_uv_ to detect shuttle vector (28), RM_p23 to detect native pRM plasmid (12), and recombinase (invertase), Hsp2, and helicase (RecD) to detect *R. amblyommatis* strain AaR/SC plasmids pRAM18, -23, and -32, respectively (12).

### PCR and sequence confirmation of rickettsial species and copy number estimates of native pRM and pRM shuttle vectors in transformants.

To confirm species identities, portions of the *ompA* gene of spotted fever group rickettsiae were amplified using primers 190-70/190-602 (41) and rickettsial genomic DNA as the template (42). PCR products were purified with the DNA Clean and Concentrator kit (Zymo Research), as per the manufacturer’s protocol, for Sanger sequencing on an ABI 3730 Excel automated sequencer (University of Minnesota Genomics Center). The sequences were compared to rickettsial *ompA* sequences in GenBank by using BLASTN (NCBI, NIH).

Three sets of specific primer pairs (Table 2) were designed for PCR amplification of native pRM, while the dGFPuvF2/R2 primer pair (Table 2) was used to amplify a product specific to pRM shuttle vectors in rickettsiae. PCRs used 100 ng of template, 1 mM mixed deoxynucleoside triphosphates (dNTPs), 0.5 μM each primer, and 1.25 U of GoTaq polymerase in 50 μL final 1× PCR buffer (Promega, Madison, WI) and were run in a Robocycler (Stratagene, La Jolla, CA) as follows: 1 cycle at 95°C for 3 min, followed by 40 cycles at 95°C for 15 s, 51°C for 30 s, and 72°C for 1 min, and then one final cycle at 72°C for 7 min.

Real-time quantitative PCR (qPCR) was used to estimate relative copy number ratios (43, 44) of single-copy genes specific to the *R. monacensis* chromosome (*gltA* encoding citrate synthase), the native pRM plasmid (RM_p5 locus for transposon resolvase), and the pRM-derived shuttle vectors (GFP_uv_) by using three primer pairs (Table 2). With the exception of a 56°C annealing temperature, reactions and copy number estimates were executed as previously described (2).
