# Supplementary material for: Examination of Rickettsial Host Range for Shuttle Vectors Based on dnaA and parA Genes from the pRM Plasmid of Rickettsia monacensis
Source: Appl Environ Microbiol. 2022 Mar 24;88(7):e00210-22. doi: 10.1128/aem.00210-22 (PMC9004397; doi:10.1128/aem.00210-22)
Supplement: Supplemental file 1 — Table S1 and Fig. S1. Download aem.00210-22-s0001.pdf, PDF file, 0.2 MB [file aem.00210-22-s0001.pdf]

SUPPLEMENTAL TABLES AND FIGURES

Table S1. Summary of plasmids found in *Rickettsia spp*.

# of plasmids	Rickettsial Species
0	*R. parkeri*, *R. akari*, *R. rickettsia*, *R. typhi*, *R. conorii*, *R. slovaca*, *R. sibirica*, *R. japonica*, *R. montanensis*, *R. prowazeckii*, *R. canadensis*, R. bellii, *R. philipii*
1	*R. peacockii*, *R. monacensis*, *R. africae*, *R. massiliae*, *R. helvetica*, *R. felis* (LSU), *R. australis*, *R. rhipicephali*
2	*R. felis* ($URRWXCal_2$)
3	*R. amblyommatis*, *R. raoultii*
4	*R. t. buchneri*

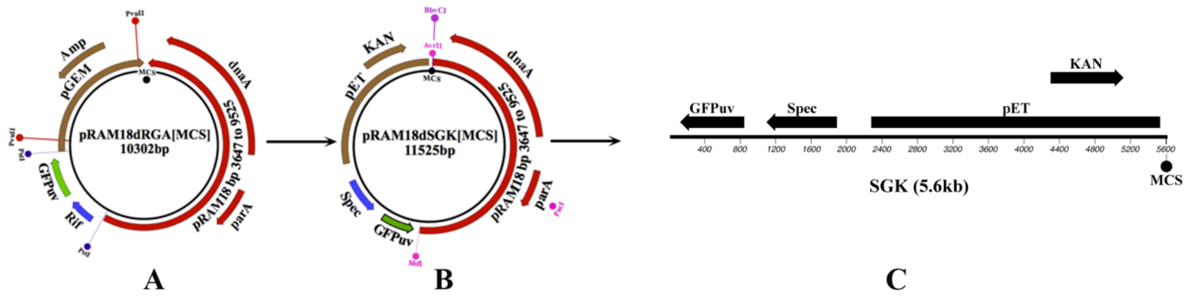

FIG S1 Construction of the selection reporter cassette used for sub-cloning pRM. A) pGEM (brown arrow) and the gfp_{uv} /Rif cassette (green and blue arrows respectively) were removed from pRAM18dRGA[MCS] with PvuII (red balloons) and PstI (purple balloons) restriction enzymes respectively. (B) pET-28a and the Spec/ gfp_{uv} cassette were inserted into the PvuII and PstI sites respectively forming pRAM18dSGK[MCS]. (C) Restriction digestion of pRAM18dSGK[MCS] with AvrII/SbfI/PacI (pink balloons) or BbvCI/SbfI/PacI (pink/purple balloons) and gel purification of fragments yielded the linear 5.6 kbp SGK fragment (Fig. 1B) into which pRM fragments were cloned to create the pRMΔ1, 2 and 3 shuttle vectors (Fig. 1D). SGK contains a multiple cloning site (MCS), the Spec/ gfp_{uv} selection reporter, and sequences conferring stable replication in *E. coli*.
